# The Diamond Jubilee and the Hospitals

**Published:** 1898-01-29

**Authors:** 


### The Institutional Workshop.

### THE DIAMOND JUBILEE AND THE HOSPITALS.

### GUY'S HOSPITAL.

With reference to the remarks made in these columns last week, the following appeared in the *Pall Mall Gazette* of January 22nd:—

"Mr. Cosmo Bonsor, M.P., treasurer of Guy's Hospital, stated to a representative of the *Pall Mall Gazette* that the writer of the article referred to was under an entire misapprehension of the position as far as Guy's was concerned. In order to open the wards a considerable capital expenditure would be necessary, details of which, added Mr. Bonsor, the superintendent, Dr. Perry, would readily supply.

"In the course of a conversation with our representative, Dr. Perry explained that in order to make use of the liberal grant the hospital had received from the Prince's Fund—and the hospital authorities fully appreciated how exceedingly liberal it was—considerable alterations would be required, and for this purpose an appeal had been made.

"'The two wards that are to be opened,' continued Dr. Perry, 'contain fifty-seven beds each. One of them has been used to provide sleeping accommodation for the forty-eight nurses who have been added to the staff, and in order to render it available it will be necessary to provide accommodation for these nurses elsewhere. Of late years the tendency has been to increase the proportion of nurses to the number of patients, and thus greater efficiency has been secured. In order to give the nurses longer hours off duty, thirty have been added to the nursing staff; ten more have had to be engaged in consequence of the reopening of the Queen Victoria ward, which is reserved for diseases of women; while an arrangement whereby we provide night nurses with a good hot midnight meal, outside the wards, has necessitated the engagement of an additional eight for relief purposes. These additions were made before the award from the Prince's Fund, and the housing of the nurses is a matter of necessity.

"'The second ward has been used—in the absence of funds to open it in the ordinary way—for the accommodation of a class of patients who pay three guineas a week for nursing and medical accommodation, in addition to fees to the surgeons or physicians attending them.

"'In regard to this ward the committee recently reported to the General Council in these words: "Your committee have every reason to believe that the ward as at present conducted satisfies a real public want by affording the benefits of the best nursing and professional skill to a class of persons who, though not actually in poor circumstances, are not well enough off to afford the expenses entailed by serious surgical operations or prolonged illness at home." We considered,' continued Dr. Perry, 'that as the ward could not be used in any other way, we were quite justified in utilising it in that manner, and thus adding £1,000 a year to the funds. And it is considered desirable to provide room for these paying patients in another part of the hospital, since, as the committee pointed out, the provision of such a ward fills a real public want.'

"Dr. Perry further declared that an extension of the laundry would be required in consequence of the reopening of the two wards referred to. It might be said, he remarked, that if the laundry was sufficient when the whole of the wards were open it ought to be sufficient now. The answer is that there is more attention paid to hygiene now; sheets and other linen are changed more frequently, and then there is the addition to the nursing staff to be taken into consideration.

"Another point to which Dr. Perry drew attention was the statement in THE HOSPITAL that £6,600 should be sufficient to cover the maintenance of the 114 new beds. According to 'Burdett's Hospital Annual' the annual cost per bed in the London Hospital is £70, though the London has 800 beds in all, and the cost per bed should be less with larger numbers. But, said Dr. Perry, the writer in THE HOSPITAL expects the beds at Guy's, where there will be only 650, when the additional number is added, to work down to a lower sum than at the London.

"The authorities at Guy's are pushing ahead with the structural alterations, and Dr. Perry showed our representative a letter he had just received from the architect stating that the builders would commence forthwith."

#### OUR VIEW.

The reply of Dr. Perry is satisfactory to the extent that it reveals how fully alive the authorities of Guy's Hospital are to the necessity of modernising and improving the older portions of the existing buildings. Thanks to Listerism, old buildings modernised are quite adequate for hospital purposes in the present day, and we hope that, like the London Hospital, the governors of Guy's may soon decide to expend at least £10,000 on brightening up the old buildings and renewing the furniture and fittings in various wards which are urgently needed. We hope that, whatever else the governors may do, they will certainly not abolish the paying patients' department, which has done an immense amount of good, and the institution of which has no doubt won for Guy's extended sympathy and increased support. Adopting Dr. Perry's figure of £70 as the annual cost per bed in the London Hospital, either the reporter has misunderstood what Dr. Perry stated or the figures quoted are inaccurate. The vacant wards at Guy's provide accommodation for 114 beds, from which number one-fifth must be deducted as the average number which will not be constantly in use throughout the year, leaving 91 beds constantly occupied. Seventy times 91 makes the total annual cost of maintaining the two vacant wards, containing 114 beds, at their full strength only £6,370, whereas Guy's Hospital has received a grant of £6,600 from the Prince of Wales's Fund. This should leave Guy's Hospital a balance of revenue to meet contingencies of about £230 per annum.

### THE METROPOLITAN HOSPITAL SUNDAY FUND.

If the criticisms levied at the Hospital Sunday and Hospital Saturday Funds by certain persons who profess an interest in hospitals and charities, upon the existence of which some of them, at any rate, depend for their livelihood, be carefully read, their meaning, if they have any meaning at all, would appear to lie in the fear which they express on the part of such persons that should these great central funds succeed in raising sufficient money to place all the voluntary hospitals upon an adequate financial basis, their occupation will be gone. Tested by any other consideration than the mere commercial aspect as it affects the individual critic, some of the criticisms are not only without knowledge, but without sense. We know that the most experienced and able of hospital officials and managers treat these criticisms with the contempt which they deserve. It may be useful, however, to call attention to them here when dealing with the effects of the Diamond Jubilee Year, because the critics have wielded their pens with greater facility, and pointed them with new venom, owing to the successful attempt to utilise the sentiment which has been aroused to secure an increasing volume of hospital support. Whilst they admit, on the one hand, that, on the whole, hospitals have experienced a steady and reliable flow of contributions; they maintain, on the other, that the more success the central funds attain to, the worse will be the condition of the hospital finances. Self-destructive criticism of this kind is highly entertaining to the initiated, but it might possibly mislead a few members of the public, if its absurdity is not made apparent. The contention is that the Sunday and Saturday Funds misuse the power of the purse which they possess. Now, how malicious and untruthful such a statement as this is has been sufficiently shown by the able papers recently read before the Hospitals Association by Sir Sydney Waterlow, the vice-president of the Hospital Sunday Fund; and Mr. R. B. D. Acland, the chairman of the Hospital Saturday Fund. Whatever may be the faults or failings of either or both these funds, no reasonable and well-informed person will for a single instant maintain, that the distribution of the Funds entrusted to the Councils of these bodies has been carried out with anything but the maximum of care, or that it does not exhibit a whole-hearted desire to do justice, and to so make the awards as to secure an adequate recognition of every institution in proportion to its efficiency, needs, and merits. The critics we have in view who maintain that they desire to increase the resources of the hospitals signally fail to bring any money into the hospital coffers. Indeed they reveal their true character whenever one or both of the Funds shows a falling-off in any year. Whenever this happens these critics clap their hands with glee, attack the Funds vigorously, and declare that both are gigantic failures, and that the hospitals would do very well without the £60,000 a year which they are the means of raising without cost to the hospitals.

Metropolitan Hospital Sunday Fund Collections, 1895, 1896, and 1897, Arranged According to Amount Collected by Each Denomination.

| Denomination. | 1895. | | | 1896. | | | 1897. | | |
|---|---|---|---|---|---|---|---|---|---|
| | £ | s. | d. | £ | s. | d. | £ | s. | d, |
| Church of England.. | 30,254 | 17 | 7 | 32,651 | 6 | 9 | 29,635 | 17 | 2 |
| Congregationalists... | 1,620 | 6 | 10 | 1,524 | 5 | 9 | 1,622 | 1 | 6 |
| Jews ... ... ... | 1,147 | 2 | 6 | 1,232 | 18 | 3 | 1,806 | 8 | 0 |
| Baptists ... ... | 915 | 18 | 1 | 942 | 19 | 5 | 866 | 5 | 7 |
| Wesleyans ... ... | 1,011 | 8 | 11 | 1,005 | 11 | 5 | 909 | 19 | 6 |
| Presbyterians ... | 1,121 | 11 | 9 | 1,186 | 0 | 2 | 1,010 | 0 | 0 |
| Roman Catholics ... | 505 | 18 | 0 | 422 | 2 | 11 | 395 | 12 | 5 |
| Unitarians ... ... | 433 | 12 | 3 | 381 | 5 | 3 | 355 | 5 | 9 |
| Society of Friends... | 136 | 11 | 11 | 116 | 19 | 5 | 102 | 2 | 1 |
| Greek Church ... | 101 | 6 | 6 | 104 | 14 | 0 | 93 | 9 | 6 |
| Swedenborgians ... | 26 | 6 | 3 | 27 | 8 | 11 | 24 | 14 | 8 |
| German Lutherans.. | 96 | 12 | 5 | 114 | 18 | 8 | 106 | 10 | 0 |
| Methodists (United Free) ... ... | 21 | 18 | 1 | 29 | 16 | 9 | 27 | 5 | 10 |
| Church of Scotland.. | 114 | 12 | 0 | 135 | 4 | 0 | 105 | 16 | 0 |
| Methodists (Primitive) ... ... | 16 | 1 | 3 | 31 | 11 | 11 | 14 | 10 | 11 |
| Reformed Episcopal Church ... ... | — | | | — | | | 39 | 2 | 1 |
| Welsh Calvinistic Methodists ... | 43 | 7 | 5 | 18 | 13 | 0 | 25 | 0 | 4 |
| Catholic Apostolic... | 77 | 10 | 3 | 83 | 16 | 11 | 61 | 5 | 1 |
| Free Church of England ... ... | 10 | 1 | 7 | 12 | 3 | 10 | 12 | 10 | 7 |
| Methodists (New Connexion) ... | 1 | 0 | 0 | 1 | 0 | 0 | 1 | 5 | 6 |
| Moravians ... ... | — | | | — | | | 2 | 17 | 0 |
| Various ... ... | 638 | 17 | 5 | 449 | 11 | 1 | 248 | 19 | 3 |
| Total ... ... | 38,295 | 1 | 0 | 40,472 | 8 | 5 | 37,373 | 19 | 10 |

This last point is interesting, because an attempt has been made to show that the slight falling-off in the total sum raised on Hospital Sunday in London in 1897 proves to demonstration that that fund is a failure, and that it has been seriously injured by the various new schemes for assisting charities which have been established to commemorate the Diamond Jubilee of the Queen. The table in the preceding column, showing the total sum raised and the amount contributed by each denomination on Hospital Sunday in the years 1895, 1896, and 1897, is worthy of close attention: It will be seen that, although the amount derived from congregational collections on Hospital Sunday in 1897 is about £3,100 less than that of 1896, it is only about £922 less than the sum paid in from this source during 1895. Practically the whole of the difference in the amounts received from congregational collections in 1897, as compared with 1896, is accounted for by a reduction to the extent of £3,000 in the sum received from the Church of England. Was this reduction due to the Diamond Jubilee, or is there any other cause which will more than account for the difference between the two years? We find on going into the details that 58 churches which made collections in 1896 have sent no sum so far to the Hospital Sunday Fund for the year 1897. The average collections of each church of England in 1896 was £30 12s., and in 1897 £29 7s. It follows that the falling-off in 1897 was therefore due to two causes; first, to 58 churches which made no collection, and thereby diminished the receipts to the extent of £1,774 16s.; secondly, that the average collection was £1 5s. per church less in 1897, which represents a total loss to the Fund of £1,261 5s. Was there any cause which affected the Church of England alone which might have produced these results? Undoubtedly there was. First of all, a party, led by Bishop Barry, made strenuous efforts to get the day for the collections changed from June 20th (Accession Day) to some other day, in order to make a general collection on the former date to help the Clergy Sustentation Fund. One or two articles appeared in the *Times* advocating this change, and in the diocese of Rochester especially we understand that this agitation exercised a certain influence on the Hospital Sunday Fund collections; and, as a matter of fact, the Clergy Sustentation Fund is reported to have received much more than the £3,000 referred to from moneys collected on June 20th last in Greater London. This is a very simple and reasonable explanation, and proves that the diminished collections were due to an exceptional cause which will not recur.

This view is confirmed by a reference to the contributions of other congregations as set forth in the table. It will there be noticed that, although the sums received from the Congregationalists' collections in 1896 were less than in 1895, they paid in a larger sum in 1897 than in either of the two former years. The Jews contributed upwards of £600 more in 1897 than in the previous years. There was a slight falling off in the sums received from the Baptists, the Wesleyans, and the Presbyterians, and a marked falling off in the collections in Roman Catholic places of worship, which seem to be steadily diminishing. At the present rate they must speedily disappear altogether unless Cardinal Vaughan through his clergy can move the congregations to exert themselves on behalf of the hospitals. The collections made by the Unitarians and the Society of Friends exhibit the same characteristics as the Roman Catholics. The contributions from the other churches are too small to enable a comparison of much value to be made, but the miscellaneous collections fell from £639 in 1895 to £450 in 1896, and to £249 in 1897. These latter figures may be due in some measure to a better and more accurate classification of the various places of worship in the latter years.

OTHER SOURCES OF REVENUE.

The total deficiency in the amount available for distribution by the Hospital Sunday Fund in 1897, as compared with 1896, amounts to £5,032. Of this sum, £3,000 may be put down to the causes already explained, £1,230 more represents a falling-off in the donations sent direct to the Mansion House, £500 of which represents half the usual sum given by Sir Savile Crossley for many years. Sir Savile, in 1897, divided his gift of £1,000 in equal moieties between the Hospital Sunday and the Prince of Wales's Funds. It will be seen, too, that the collections in 1897 were only £922 less than those of 1895. Finally, there were no legacies at all paid to the Hospital Sunday Fund during 1897, and hospital managers know full well that all charities are liable to great fluctuations from this source. It is satisfactory to find that the more fully the facts are ascertained, and the closer the examination to which they are submitted, the more certain does it become that the falling-off in the amount raised by the Metropolitan Hospital Sunday Fund during 1897 as compared with 1896 is due to special causes which have little or no bearing upon the Diamond Jubilee collections in favour of the Prince of Wales's Hospital Fund and other movements established to commemorate the 60th year of Her Majesty's reign. We are aware that a certain number of institutions, which labour, no doubt, under the mistaken idea that such statements are calculated to attract and win public sympathy, have put forward pleas to the effect that they have been seriously crippled in income and permanently damaged financially as the result of the special efforts made during 1897. Having taken some pains to ascertain the facts, we are led to the conclusion that all such statements should be received with scepticism, and that none of them should be believed unless they are supported by financial statements based upon accurate data duly vouched by competent and independent auditors.

